# Orphan Crops: A Best Fit for Dietary Enrichment and Diversification in Highly Deteriorated Marginal Environments

**DOI:** 10.3389/fpls.2022.839704

**Published:** 2022-02-24

**Authors:** Abidemi Olutayo Talabi, Prashant Vikram, Sumitha Thushar, Hifzur Rahman, Hayatullah Ahmadzai, Nhamo Nhamo, Mohammed Shahid, Rakesh Kumar Singh

**Affiliations:** International Center for Biosaline Agriculture (ICBA), Dubai, United Arab Emirates

**Keywords:** orphan crops, marginal environments, climate change, food and nutritional security, improved livelihood

## Abstract

Orphan crops are indigenous and invariably grown by small and marginal farmers under subsistence farming systems. These crops, which are common and widely accepted by local farmers, are highly rich in nutritional profile, good for medicinal purposes, and well adapted to suboptimal growing conditions. However, these crops have suffered neglect and abandonment from the scientific community because of very low or no investments in research and genetic improvement. A plausible reason for this is that these crops are not traded internationally at a rate comparable to that of the major food crops such as wheat, rice, and maize. Furthermore, marginal environments have poor soils and are characterized by extreme weather conditions such as heat, erratic rainfall, water deficit, and soil and water salinity, among others. With more frequent extreme climatic events and continued land degradation, orphan crops are beginning to receive renewed attention as alternative crops for dietary diversification in marginal environments and, by extension, across the globe. Increased awareness of good health is also a major contributor to the revived attention accorded to orphan crops. Thus, the introduction, evaluation, and adaptation of outstanding varieties of orphan crops for dietary diversification will contribute not only to sustained food production but also to improved nutrition in marginal environments. In this review article, the concept of orphan crops vis-à-vis marginality and food and nutritional security is defined for a few orphan crops. We also examined recent advances in research involving orphan crops and the potential of these crops for dietary diversification within the context of harsh marginal environments. Recent advances in genomics coupled with molecular breeding will play a pivotal role in improving the genetic potential of orphan crops and help in developing sustainable food systems. We concluded by presenting a potential roadmap to future research engagement and a policy framework with recommendations aimed at facilitating and enhancing the adoption and sustainable production of orphan crops under agriculturally marginal conditions.

## Introduction

The current world population is 7.8 billion, which is projected to reach 9.7 billion by 2050 (United Nations Population Fund, 2019). Tilman et al. (2011) indicated that agricultural production must increase by 60–110% to meet the global requirement of the projected population by 2050. Potapov et al. (2021) estimated total global cropland area in 2019 to be 1,244 Mha, along with a corresponding total annual net primary production of 5.5 Pg (5.5 × 10^15^) carbon year^–1^. Arable lands are usually affected adversely by land degradation and climate change, which have become the present-day reality of our ecosystem. Soil and water salinity are also major contributors to the decline in productivity of agricultural lands, thereby limiting food and fodder production (Khan et al., 2006). About 1,125 million hectares of land are affected by salinity across the globe, with human-induced salinization accounting for about 76 million hectares (Wicke et al., 2011, 2020). As of now, an estimated one-fifth of the land area under irrigation is adversely affected by salinity while the current rate of advancement in salinization is projected to render about 1.5 million hectares of land useless every year (Hossain, 2019). Water scarcity also constitutes a limitation to the intensive use of land. According to White (2014), water scarcity occurs when water is grossly inadequate to meet human and ecosystem requirements simultaneously. This scenario is often occasioned by physical water scarcity or lack of required infrastructure for the provision of access to available water resources (economic scarcity). Natural phenomena such as aridity and drought could cause physical water scarcity; however, human influences, including desertification and water storage, could also trigger physical water scarcity (Pereira et al., 2009; White, 2014). In view of the present scenarios, farmers are forced to grow their crops in saline soils under rainfall deficit and drought and/or heat stress during the cropping season. Interestingly, some underused alternative crops (e.g., foxtail millet, proso millet, Bambara groundnut, and barnyard millet, among others) have a competitive nutritional profile comparable to and in some cases better than that of commonly grown food crops such as maize, rice, and wheat. These crops not only have high concentrations of essential nutrients but also have potential for outstanding performance under suboptimal growing conditions (Bharucha and Pretty, 2010).

To mitigate the impact of climate change and continued land degradation as well as to increase/sustain global food production, two potential approaches can be pursued. The first involves increasing yield per unit area of crops through genetic enhancement and improved agronomic practices (Parry and Hawkesford, 2010). The second option involves bringing back degraded lands into productive use through the introduction, testing, and adaptation of alternative nutrient-dense and stress-tolerant crops to marginal environments especially where these crops have not been previously cultivated (Mabhaudhi et al., 2019). This would facilitate dietary diversification and pave the way for food self-sufficiency in the affected regions. In summary, to achieve sustainable food and nutrition security, there is a need to breed crops for stress tolerance and resource use efficiency in marginal environments as well as devote more research attention toward the introduction, evaluation, and adaptation of underused crops for dietary diversification.

This review was conducted to examine the potential of some selected orphan or underused crops for diversified and balanced diets, improved food and nutrition security, income generation by rural poor, empowerment of women and youth, as well as salvaging degrading marginal environments. The potency of modern breeding strategies (including high-throughput genotyping and phenotyping, marker-assisted selection, genomic selection, gene editing, mutation breeding, and other approaches that could fast-track the genetic enhancement of these crops) along with the anticipated impact of dietary diversification on the social fabric are also highlighted along with a potential roadmap for future research engagement and a policy framework.

### Marginality

The concept of marginal environments is subjective and therefore defined from diverse perspectives, including societal, infrastructure, health, education, political, economic, environmental, and development index (Gurung and Kollmair, 2005). In the context of agriculture, we consider marginal environments as lands that are limiting in the provision of optimal conditions required for crop growth and productivity as well as those that result in poor economic returns when used for crop cultivation. A comprehensive definition of marginal environments was provided by Pender and Hazell (2000), who referred to these environments as less-favorable agricultural areas (LFAAs) characterized by constrained agricultural potential and resource degradation attributable to biophysical and politico-socioeconomic factors. The authors indicated that the low production potential of these LFAAs is driven by rugged terrains, extreme weather conditions, poor soil and water quality, lack of socioeconomic connectivity, and limited exposure to agricultural intensification opportunities. They also added that drought and erratic rainfall, salinization, and other factors present significant constraints for intensive agriculture in LFAAs.

### Orphan Crops

According to Dawson et al. (2007), orphan crops are those that have either originated from a geographical location or become “naturalized” due to many decades (usually greater than 10 decades) of cultivation alongside natural selection and/or artificial selection by farmers. The term “orphan” is derived from the state of neglect and abandonment of the crops by the scientific community despite their grossly underexploited food and nutritional potential that can contribute to food and nutrition security, healthy living, improved livelihood of farmers, and improvement of the environment (Tadele and Assefa, 2012; Tadele and Bartels, 2019). Orphan crops are also referred to as underused, lost, indigenous, minor, promising, or future crops (Tadele, 2019). Orphan crops have a history with indigenous people and are generally accepted among the rural populace for their nutritional and health values as well as adaptation to prevailing local stresses and growing conditions (Kamei et al., 2016). With renewed awareness of the potential of orphan crops in terms of being nutrient-dense, amenable to diverse food systems, and tolerant of suboptimal growing conditions, research attention is beginning to shift in the direction of these crops, for which the knowledge of indigenous people would be invaluable.

Although number of orphan crops are native to different continents or regions like Africa, Asia, South America, North America, South Pacific, and Australia, among others (Davies et al., 2005; Foyer et al., 2016; Mueller et al., 2017; Kagawa-Viviani et al., 2018; Dawson et al., 2019; Kehinde et al., 2022), but a plant species may be called orphan for one particular region may not be necessarily orphan crop for others. Several orphan crops listed for Africa include crops that are commonly grown in South Asia and other parts of the world. For example, the African Orphan Crops Consortium (AOCC) listed okra, onion, cashew, custard apple, jack fruit, papaya, watermelon, coconut, pumpkin, finger millet, sweet potato, lentil, mango, bitter gourd, drumstick, mulberry, banana, guava, etc., as part of the 101 most important orphan crops in Africa^1^, whereas these crops are widely cultivated in other areas of the world and are not considered as orphan crops in those regions. In the past couple of decades, crop species from different parts of the world have been gaining attention in other regions, that is, outside of their niche. Quinoa (*Chenopodium quinoa*) is one of the most captivating cases, moving out of South America (Bolivia, Peru, and Chile) and expanding globally. Quinoa varieties have been bred specifically for the European and North American environments (Alandia et al., 2020). Similarly, amaranth, buckwheat, millets, and other crops have gained attention outside of their centers of origin and domestication (Rodríguez et al., 2020).

It is noteworthy that the AOCC selected the 101 priority orphan crops based on three primary criteria: (1) being rich in micro- and macronutrient contents, (2) being relevant to Africa, and (3) having a need for developing breeding resources (Hendre et al., 2019). In a similar manner, the Food and Agriculture Organization of the United Nations Regional Office for Asia and the Pacific (FAO/RAP) identified and designated traditional underused crops as Future Smart Food (FSF) based on four criteria: high nutritional profile, climate resilience, local availability, and economic viability. This was in line with the FAO/RAP Initiative for Zero Hunger (Li and Siddique, 2018). Thus, in this review, we identified 13 orphan crops based on four criteria: (1) resilience to salinity, drought, and/or heat stress, which are prevalent in marginal environments; (2) high nutritional profile; (3) amenability to diverse cropping systems; and (4) local availability for economic growth and social development. These crops were assigned to three categories: cereals and pseudo-cereals, pulses, and oil crops (Table 1).

**TABLE 1:** Selected nutrient-dense orphan crops for dietary diversification in marginal environments.

S. no.	Crop	Scientific name	Country/region of origin	Reaction to stresses^†^			Importance	Source of information
				Drought	Salinity	Heat		
(1)	Finger millet	*Eleusine coracana*	East Africa	T	MT	–	Rich in methionine, High content of fiber and minerals	National Research Council, 1996; Chandrashekar, 2010; Bhatt et al., 2011; FAO, 1995
(2)	Proso millet	*Panicum miliaceum*	Egypt and Arabia	T	T	T	Rich in protein, fiber, B vitamins, and minerals	FAO, 1995; Kalinova and Moudry, 2006
(3)	Barnyard millet	*Echinochloa* spp.	Central Asia	T	T	–	Source of high protein, fiber, high iron, and gluten-free	Saleh et al., 2013
(4)	Buckwheat	*Fagopyrum tataricum*	China	T	S	S	Low-gluten, high protein content, rich in vitamin B, and has nutraceutical properties	Wu et al., 2019; Pirzadah and Rehman, 2021
(5)	Fonio	*Digitaria* sp.	West Africa	T	MT	MT	Gluten-free, rich in the amino acids, cysteine, and methionine. Matures within 60–70 days.	Temple and Bassa, 1991; National Research Council, 1996
(6)	Little millet	*Panicum sumatrense*	India	T	MT	T	Low in calories but rich in dietary fiber, magnesium, bioactive compounds, and other essential minerals and vitamins.	Itagi et al., 2013; Ajithkumar and Panneerselvam, 2014
(7)	African yam bean	*Sphenostylis stenocarpa*	West Africa	T	–	–	Abundant in protein, dietary fiber, carbohydrate, and minerals	Baiyeri et al., 2018; Anya and Ozung, 2019; George et al., 2020
(8)	African winged bean	*Psophocarpus tetragonolobus*	New Guinea and Indonesia	–	–	T	Rich in dietary protein and low in anti-nutritional factors	Wan Mohtar et al., 2014; Mohanty et al., 2015; Vatanparast et al., 2016
(9)	Moth bean	*Vigna aconitifolia*	India	T	MT	T	Rich in protein and minerals such as Ca, Mg, K, Zn, and Cu	Gupta et al., 2016; Tiwari et al., 2018; van Zonneveld et al., 2020
(10)	Bambara nut	*Vigna subterranea*	West Africa	T	MT	T	Rich in quality protein and dietary fiber. Also, a good source of calcium, phosphorus, iron, and vitamin C.	National Research Council, 1996; Feldman et al., 2019; Soumare et al., 2021; Olanrewaju et al., 2022
(11)	Jatropha	*Jatropha curcas*	African tropics	T	T	MT	Rich source of protein and oil	King et al., 2009; Maes et al., 2009; Devappa et al., 2010
(12)	Jojoba	*Simmondsia chinensis*	Northern Mexico and Southwestern United States	T	T	T	Oil makes 50% of seed by weight, contains 97% monoesters of long-chain fatty acids giving it very long shelf life	Dunstone and Begg, 1979; Benzioni and Dunstone, 1986; Makpoul et al., 2017
(13)	Camelina	*Camelina sativa*	Eastern Himalayas, China, Japan, and Malaysia	T	MT	–	High level (about 45%) of omega-3 fatty acids	Acamovic et al., 1999; Bakhshi et al., 2021
(14)	Teff	*Eragrostis tef*	Ethiopia	T	MT	T	Gluten free and highly rich in iron and other key nutrients	National Research Council, 1996; Aptekar, 2013; Zhu, 2018; Gelaw and Qureshi, 2020

*^†^T, tolerant; MT, moderately tolerant; S, sensitive.*

It suffices to indicate that the intention is not for orphan crops to dominate the diet or compete with the major food crops but to complement production to meet the food requirements of the fast-increasing population as soon as possible. Orphan crops are also outstanding in performance and could thrive better than major food crops in environments where they are indigenous and widely cultivated. Promotion and adoption of orphan crops are expected to achieve direct and indirect impacts on many global challenges.

## Potential and Prospects of Orphan Crops in Food Systems

Despite being grown on infertile portions of farms and commonly cultivated in marginal agricultural regions; orphan crops continue to play a significant role in food security. They provide the calorie requirement of people living in areas where major food crops such as maize, rice, and wheat cannot be produced optimally. It is also noteworthy that orphan crops require low inputs by nature; thus, farmers will be spending far lesser amount of money on production compared to those of major food crops.

### Role in System Diversification, Sustainability, and Soil Health

While maize, rice, and wheat dominate cropping systems; these crops do not perform well in marginal areas. Orphan crops can be grown successfully as sustainable alternatives to satisfy the calorie requirements of communities. The use of orphan crops to occupy niches in production in multiple cropping systems has several advantages. For instance, niche management using alternative orphan crops addresses the food production challenges in locations where climate and other environmental factors can no longer support traditional cereals. Second, selected orphan crops can be used to explore sustainable crop diversification opportunities for multiple cropping, thereby supporting yield stability, building up of disease and pest resistance, higher resource use efficiency, and intensification where conditions allow for this. Local crop intensification and the use of diverse crop cycles have been put forward as a solution to improve food security without increasing the area under cultivation subject to site-specific productivity and actual environmental costs (Waha et al., 2020). As the frequency of climate extremes increases, mixed crop production practices that include wide adaptation traits are important for maintaining food security (Iijima et al., 2016). Therefore, the inclusion of orphan crops can increase both food security and opportunities for designing sustainable crop intensification strategies. Alternative crops in the form of orphan crops score highly in all areas of the four pillars of food security: access, availability, use, and stability. In particular, the majority fit in sustainable production practices and support dietary diversity. Orphan crops could have a different supplemental requirement (fertilizers/minerals) for their growth and development as compared with the staple cereals that largely dominate food systems, but definitely not exhaustive requirements. Soil nutritional status affects the nutritional profile of crop produce (Rekik, 2020). Therefore, it is imperative to work out soil health needs and define specific fertilizer requirements prior to including orphan crops in food systems.

Nutritional requirements significantly affect food systems. Small millets, which are mostly orphan crops, play significant roles in the diets and nutrition of local indigenous people in Africa, Asia, and South America. Compared with traditional cereals, orphan crops such as finger, foxtail, and proso millet, among others, have high potential and value as food sources as they provide gluten-free flour, are higher in dietary fiber and essential amino acids (leucine, isoleucine, and lysine), and have good lipid content (Dias-Martins et al., 2018). For example, finger millet has comparable carbohydrate, protein, and fat content as wheat and rice, but the crop is by far the richest source of calcium (300–350 mg/100 g), as detailed in Table 2A. Finger millet also has comparable and, in some cases, higher essential amino acids, vitamins, and micronutrients than wheat and rice (Table 2B). Other small millets also serve as abundant sources of phosphorus and iron. In a study conducted by Devisetti et al. (2014), the protein content of foxtail and proso millets was found to be high (e.g., 14.8 g/100 g), and measured total and soluble dietary fiber was significantly high. In the brown and whole grains, both small grains had significant phenolic compounds and phytic acid, which can be overcome by processing, while the nitrogen solubility of their flours was good (e.g., up to 16.4 mg/g) in water (Devisetti et al., 2014). Work of Das et al. (2019) has shown that proso millet, for example, provides vitamins (niacin, B-complex vitamins, and folic acid), minerals (P, Ca, Zn, and Fe), and essential amino acids (methionine and cysteine). Proso millet also has comparable contributions to protein, energy, and carbohydrates.

**TABLE 2A:** Nutritional profile of selected millets compared with those of wheat and rice.

Nutrient composition												
**Crop**	**Carbohydrates (g)**	**Protein (g)**	**Fat (g)**	**Energy (KCal)**	**Crude fiber (g)**	**Mineral matter (g)**	**Ca (mg)**	**P (mg)**	**Fe (mg)**			
Finger millet	72	7.3	1.3	328	3.6	2.7	344	283	3.9			
Foxtail millet	60.9	12.3	4.3	331	8	3.3	31	290	2.8			
Proso millet	70.4	12.5	1.1	341	2.2	1.9	14	206	0.8			
Barnyard millet	65.5	6.2	2.2	307	9.8	4.4	20	280	5			
Little millet	67	7.7	4.7	341	7.6	1.5	17	220	9.3			
Pearl millet	67.5	11.6	5	361	1.2	2.3	42	296	8			
Wheat (whole)	71.2	11.8	1.5	346	1.2	1.5	41	306	5.3			
Rice (raw, milled)	78.2	6.8	0.5	345	0.2	0.6	10	160	0.7			
Teff	73.13	13.3	0.4	367	8	–	180	429	7.63			

**Essential amino acids**												

**Crops**	**Arginine (mg/g of N)**	**Histidine (mg/g of N)**	**Lysine (mg/g of N)**	**Tryptophan (mg/g of N)**	**Phenyl Alanine (mg/g of N)**	**Tyrosine (mg/g of N)**	**Methionine (mg/g of N)**	**Cystine (mg/g of N)**	**Threonine (mg/g of N)**	**Leucine (mg/g of N)**	**Isoleucine (mg/g of N)**	**Valine (mg/g of N)**

Finger millet	300	130	220	100	310	220	210	140	240	690	400	480
Foxtail millet	220	130	140	60	420	–	180	100	190	1040	480	430
Proso millet	290	110	190	50	310	–	160	–	150	760	410	410
Barnyard millet	270	120	150	50	430	–	180	110	200	650	360	410
Little millet	250	120	110	60	330	–	180	90	190	760	370	350
Pearl millet	300	140	190	110	290	200	150	110	140	750	260	330
Wheat (whole)	290	130	170	70	280	180	90	140	180	410	220	280
Rice (raw, milled)	480	130	230	80	280	290	150	90	230	500	300	380

*(Source: Nutritive value of Indian foods, NIN, 2007 ; MILLET in your Meals, http://www.sahajasamrudha.org/ and https://data.nal.usda.gov/dataset/composition-foods-raw-processed-prepared-usda-national-nutrient-database-standard-reference-release-27).*

**TABLE 2B:** Nutritional profile of selected millets compared with those of wheat and rice.

Vitamins										
Millet	Thiamin (mg)	Niacin (mg)	Riboflavin	Vit A (carotene) (mg/100 g)	Vit B6 (mg/100 g)	Folic Acid (mg/100 g)	Vit B5 (mg/100 g)	Vit E (mg/100 g)		
Finger millet	0.42	1.1	0.2	42	–	18.3	–	22		
Foxtail millet	0.59	3.2	0.1	32	–	15	0.8	31		
Proso millet	0.41	4.5	0.3	0	–	–	1.2	–		
Barnyard millet	0.33	4.2	0.1	0	–	–	–	–		
Little millet	0.3	3.2	0.1	0	–	9	–	–		
Sorghum	0.38	4.3	0.2	47	0.21	20	1.3	12		
Pearl millet	0.38	2.8	0.2	132	–	45.5	1.1	19		
Wheat (whole)	0.41	5.1	0.1	64	0.57	36.6	–	–		
Rice (raw, milled)	0.41	4.3	0	0	–	8	–	–		
Teff	0.39	3.36	0.27	0	0.46	–	0.94	0.08		

**Micronutrients**										

**Finger millet**	**Mg (mg/100 g)**	**Na (mg/100 g)**	**K (mg/10 0g)**	**Cu (mg/100 g)**	**Mn (mg/100 g)**	**Mb (mg/100 g)**	**Zn (mg/100 g)**	**Cr (mg/100 g)**	**Su (mg/100 g)**	**Cl (mg/100 g)**

Foxtail millet	137	11	408	0.47	5.49	0.102	2.3	0	160	44
Proso millet	81	4.6	250	1.4	0.6	0.07	2.4	0	171	37
Barnyard millet	153	8.2	113	1.6	0.6	–	1.4	0	157	19
Little millet	82	–	–	0.6	0.96	–	3	0.1	–	–
Sorghum	133	8.1	129	1	0.68	0.016	3.7	0.2	149	13
Pearl millet	171	7.3	131	0.46	0.78	0.039	1.6	0	54	44
Wheat (whole)	137	10.9	307	1.06	1.15	0.069	3.1	0	147	39
Rice (raw, milled)	138	17.1	284	0.68	2.29	0.051	2.7	0	128	47
Rice	90	–	–	0.14	0.59	0.058	1.4	0	–	–
Teff	184	12	427	0.81	9.24	–	3.63	–	–	–

*(Source: Nutritive value of Indian foods, NIN, 2007; MILLET in your Meals, http://www.sahajasamrudha.org/ and https://data.nal.usda.gov/dataset/composition-foods-raw-processed-prepared-usda-national-nutrient-database-standard-reference-release-27).*

### Agricultural Systems for Health

Orphan crops support sustainable production practices of diverse cropping systems, have high feed value in livestock and fisheries, and contribute to soil health. They also contribute to net-carbon sequestration (Toensmeier et al., 2020), a goal important for climate adaptation and mitigation. These system attributes are fundamental in pursuit of good human health as supported by agriculture and related ecosystem services. The nutritional status of the crops can be considered as an overlap between agriculture and health. In this process, system performance and the ability to provide nutritious food become a “central dogma.” It is also evident that evolution of a few dominant crops largely contributes toward a risk of malnutrition and dietary diseases. Here, we give some examples of direct health benefits from agricultural products beyond nutrition. For instance, the low glycemic index of their flour products has been found to decrease the risk of type-2 diabetes mellitus and cardiovascular disease in human adults (Das et al., 2019). Foxtail millet products have been found to contribute to human health through low glycemic index and hypolipidemic and antioxidant attributes (Sharma and Niranjan, 2018). Several diseases in humans (i.e., cancer, cardiovascular disease, dental disease, diabetes, obesity, and osteoporosis) are on the increase in developed and developing countries and diets supported by orphan crops are an extremely important consideration for diminishing these diseases. The prominence of diverse foods that include orphan crops has potential to decrease the impact of epidemics from diet-driven non-communicable diseases (Watkins and Daignault, 2020; Åhlberg, 2021; Carazo et al., 2021).

### Source of Livelihood for Resource-Poor Farmers

Indigenous communities living in remote areas often grow orphan crops for their food and livelihood development (Kuhnlein et al., 2013). The proliferation of orphan crops in remote areas is driven by low adoption of modern farming methods because of limited access to agricultural services and a disconnect from input and output markets. For these indigenous communities, orphan crops are central to religious beliefs, rituals, and customary practices. These crops have also been used to develop recipes for dishes served on special occasions, in the cure of disease bouts, and as currency for the barter system of exchange for other commodities. Together with wild fruits and vegetables collected from forests, orphan crops constitute a source of the entire livelihood system for poor rural people living in remote areas.

### Consumer Taste Satisfaction

With higher incomes, a spin-off from improved literacy and rural-urban migration, most of the youth do not consume traditional dishes (Baada et al., 2019; Marchetti et al., 2020). In urban centers, consumer tastes are shaped by increased access to meat products and other dairy products at the expense of products from orphan crops (Akinola et al., 2020; Lillford and Hermansson, 2021). Large well-developed commodity value chains dictate consumption of rice, maize, and wheat products at the expense of orphan crops in urban centers (Reardon, 2015). Generational differences have not supported the use of orphan crops on a large scale. Orphan crops have maintained the diversity of food chains although they have limited taste preferences.

Some of the challenges could be as follows:

#### Relatively Inferior Yields

Most orphan crops have not been genetically improved and hence have yield lower than that of the main food crops. Attributes such as high nutrient density are lost in the poor yield and poorly applied management practices. Without improved high-yielding varieties and a positive response to management and inputs, orphan crops face extinction because of anthropogenic negligence (Azam-Ali et al., 2021).

#### No Favorable Policy Supports

Orphan crops have not received fair and supportive policy coverage worldwide. Without policies, the knowledge vested in, and cultural importance associated with orphan crops have dwindled over time (Azam-Ali et al., 2021), a situation that needs redress. Promotion and widescale popularization have been suggested to protect the huge value of orphan crops.

## Current Status of the *ex situ* and *in situ* Conservation of Selected Orphan Crops in Genebanks

The rich diversity that exists in most of the orphan crops is threatened with extinction unless their germplasm is conserved and fully characterized (Bhattacharjee et al., 2009). There have been various efforts (Sogbohossou et al., 2018; Daley et al., 2020) to conserve a few orphan crops, but, in most cases, the germplasm collections are not optimum and lack full genetic characterization. Both *in situ* and *ex situ* germplasm collections of different orphan crops will be required, followed by the full characterization of the collections to facilitate further genetic improvement efforts. At present, approximately 7.4 million accessions of different crops are stored in approximately 1,750 genebanks (FAO, 2010) around the world. Most of this genetic diversity (∼80%) belongs to the major crops and their relatives. Keeping in view the importance of orphan crops in agricultural diversification, the genebank at the International Center for Biosaline Agriculture has assembled and conserved several accessions of various orphan crops from different countries. These accessions are available for use in research and development programs for the region and other parts of the world.

Globally, *ex situ* collections of small millets and pseudo-cereals are being preserved in more than 150 genebanks across the world, of which the International Crop Research Institute for the Semi-Arid Tropics (ICRISAT) in India; Institute of Crop Germplasm Resources, Chinese Academy of Agricultural Sciences (ICGR-CAAS), in Beijing, China; Plant Genetic Resources Conservation Unit, Southern Regional Plant Introduction Station, University of Georgia, USDA-ARS, United States; and National Bureau of Plant Genetic Resources (NBPGR), New Delhi, India, are the major repositories for these orphan crops. Table 3 details the proportion of germplasm accessions of selected orphan crops under the different germplasm storage types, while the list of selected orphan crops conserved in different genebanks and the status details of the collections appear in Supplementary Table 1 (Genesys, 2017).

**TABLE 3:** Germplasms of selected orphan crops conserved under different types of germplasm storage.

Crop	Long term seed collection (%)	Medium-term seed collection (%)	Short term seed collection (%)	Seed collection (%)	Cryopreserved seeds (%)	Field collections (%)	DNA collection (%)	Others/Not specified (%)	Source of information
Finger millet (*Eleusine coracana*)	36.47	37.68	0.07	7.29			0.03	18.46	GENESYS
Proso millet (*Panicum miliaceum*)	24.34	8.15	3.53	60.75	0.005	0.33		2.90	
Barnyard millet (*Echinochloa* spp.)	29.62	31.73		19.75	0.16		0.08	18.66	
Buckwheat millet (*Fagopyrum* spp.)	31.96	7.61		42.41		0.18		17.84	
Fonio (*Digitaria* sp.)	32.86	21.43	1.43	37.14				7.14	
Little millet (*Panicum sumatrense*)	40.32	40.58	0.09	18.84				0.17	
African yam bean (*Sphenostylis stenocarpa*)	10	49.15	0.1	37.87				2.87	
African winged bean (*Psophocarpus tetragonolobus*)	4.14	5.56	0.13	24.97		0.13		65.07	
Amaranthus (Amaranth spp.)	31.14	9.10	1.88	36.94	0.05	0.02		20.87	
Moth bean (*Vigna aconitifolia*)	23.05	3.17	2.02	38.04		0.29		33.43	
Bambara nut (*Vigna subterranea*)	30.01	31.59		35.13				3.27	
Jatropha (*Jatropha curcas*)	5.86	18.05	0.16	0.81		74.31	0.81		
Jojoba (*Simmondsia chinensis*)	1.82	1.21	1.21	63.97		11.13		20.66	
Camelina (*Camelina sativa*)	57.22	18.63		15.56		0.68	7.91		
Castor bean (*Ricinus communis*)	18.36	23.89		52.78	0.02	0.85		4.10	
Teff (*Eragrostis tef*)	3.01	0.42		31.50				65.07	

## Advances in Genetic Improvement of Orphan Crops

A global-level coordinated effort is urgently required in orphan crop research that can address the array of upcoming challenges, including climate resilience, shrinking agricultural resources, hidden hunger, food/nutrition security, etc. It is noteworthy here that the Green Revolution’s genetic gain (Evenson and Gollin, 2003) has been attributed to germplasm conservation, characterization, and trade (Pingali, 2012). To achieve a successful orphan crop improvement program, both *in situ* and *ex situ* germplasm collections will be necessary, followed by their intensive characterization. The CGIAR institutes IITA and CIAT-Bioversity International and several other international agencies are making efforts^2^ (Padulosi et al., 2021); however, more organizations and intensive endeavors are needed. The One CGIAR platform has the potential to contribute significantly toward this as it is in the vicinity of several centers of crop diversity, for example, CIMMYT (Mexico), CIP (Peru), CIAT (Colombia), IITA (Nigeria), ICRISAT (India), and ICARDA (Morocco). These centers have mandatory crops and target environments. However, if they contribute toward orphan crop collection, conservation, and characterization in their target environments using their already established infrastructure, this could be quite useful.

The ease of hybridization in the staple food crops (rice, wheat, and maize) rendered them fit candidates for crop improvement programs globally. Orphan crops have varying floral biology. The tiny intricate flowers in orphan crops such as finger millet (Hilu and De Wet, 1980) and amaranth (Stetter et al., 2016) hinder crossing effectively. Further, the invariable outcrossing percentage in orphan crops, including amaranth (up to 49%), African eggplant (up to 30%), and several others, hindered hybridization (Adeniji et al., 2012). Unlike amaranth and African eggplant, other orphan crops such as grass pea (predominantly autogamous) and water yam (dioecious) facilitate the breeding process (Hanson and Street, 2008; Ghorbel et al., 2014). A focused and coordinated effort is urgently required in understanding the possibility for hybridization while addressing crossing barriers of different orphan crops.

Genetic variability in progenies from interspecific crosses is always inevitable, although few of them are viable. With success achieved through interspecific hybridization between *Dioscorea alata* (water yam), and *Dioscorea nummularia* (yam), Lebot et al. (2019) encourage replication of this strategy in other orphan crop species and suggest further opportunities to introgress superior traits. On the one hand, ample genetic variation can be created while introgressing useful traits from one species into another. Large-scale crossing followed by embryo rescue could help in opening new avenues in interspecific hybridization of relatively unexplored orphan crop species. Well-established and standardized breeding protocols, breeding schemes, and strategies for orphan crops are instrumental in crop genetic improvement.

Apart from hybridization barriers, time incurred in the generation advancement process poses another bottleneck in fast-tracking varietal improvement. Chiurugwi et al. (2019) have reviewed different technologies that have potential to transform and speed up the genetic improvement process (breeding) for orphan crops. Shortening of the crop cycle through decreasing days to flowering is the key step in this process. This can be achieved successfully by modifying growth conditions, including photoperiod adjustments through light exposure, modifying relative humidity and temperature in growth chambers, and carrying out early seed harvest (O’Connor et al., 2013; Ghosh et al., 2018). Doubled haploidy offers an alternative approach toward fast-track fixing of useful alleles in breeding germplasm (Chaudhary et al., 2019). Protocols for speed breeding have been optimized in staple cereal crops, including rice and wheat (Ohnishi et al., 2011; Alahmad et al., 2018). Speed breeding protocols are being developed in orphan crops such as grain amaranth, grass pea, and quinoa (Stetter et al., 2016; Ghosh et al., 2018). This strategy therefore offers a promising approach in addressing the breeding bottlenecks of orphan crops.

For traits *per se*, the breeding strategy of orphan crops could be slightly different from that of staple food crops for which the focus is mainly on yield. The domestication syndrome traits in orphan crops are a priority objective that facilitates their adaptation into relatively newer environments and agricultural systems. Furthermore, improved processability needs to be focused on for orphan crop products, which depends largely on their physical properties (e.g., amylose to amylopectin in cereal starches) and chemical properties (e.g., anti-nutritional phytates in legumes) (Jamnadass et al., 2020). To enhance favorable crop-crop interactions, orphan crops must be better integrated into existing farming systems by concentrating on features such as plant architecture and phenology. Fixing these traits will promote the suitability of orphan crops in relatively newer agricultural systems; thereafter, breeding programs can target yield or other economically relevant traits.

Genetic improvement of orphan crops has been achieved by landrace selection to improve domestication syndrome features, including non-shattering seed type, increased seed size and weight, lesser dormancy period, etc., in addition to imparting tolerance for biotic and abiotic stresses (Sseremba et al., 2018a,b). Targeted improvements for domestication syndrome traits using genome editing with CRISPR-Cas9 approaches have been suggested (Lemmon et al., 2018; Tripathi et al., 2019; Zaman et al., 2019), but poorly developed genomic resources pose challenges. Standardized genetic transformation protocols, tissue culture methods, well-annotated genome sequences, and other similar resources/methods have become bottlenecks in using genome editing and other transformation methods in orphan crop improvement. An integrated breeding approach focusing on domestication syndrome traits together with processability, harvestability, architecture, and phenology should help in establishing effective integrated production (intercrop) systems aided by orphan crops.

Despite the important roles of orphan crops in global food and nutrition security and their potential contribution to sustainable food production under the changing climatic scenario, the research efforts made on genetic improvement of these crops are grossly inadequate. Advances in next-generation sequencing, high throughput genotyping and phenotyping techniques have made feasible the rapid identification of yield and yield-related QTLs in these orphan crops. Subsequently, the identified QTLs can be introgressed into the desired genotypes of the orphan crops using marker-assisted breeding. After sequencing of the first orphan genome (i.e., foxtail millet) (Bennetzen et al., 2012; Zhang et al., 2012), the genomes of several other orphan crops have been sequenced: teff (Cannarozzi et al., 2014), pearl millet (Varshney et al., 2017a), finger millet (Hittalmani et al., 2017; Hatakeyama et al., 2018), broomcorn millet (Shi et al., 2019; Zou et al., 2019), wild relatives of barnyard millet (*Echinochloa crus-galli* and *Echinochloa oryzicola*) (Guo et al., 2017; Ye et al., 2020), fonio millet (Abrouk et al., 2020), adlay (Guo et al., 2020), and wild *Coix* (*C. aquatica*) (Kang et al., 2020; Liu et al., 2020). Apart from these, Chang et al. (2019) published the genome assembly of five orphan crops: Bambara groundnut (*Vigna subterranea*), lablab (*Lablab purpureus*), white acacia (*Faidherbia albida*), marula (*Sclerocarya birrea*), and moringa (*Moringa oleifera*). During the past decade, the AOCC (see Text Footnote 1) took the initiative to sequence the genome of 101 African orphan crops and re-sequencing 100 lines of each genome will speed up molecular breeding in these crops by developing better suited genotypes with higher yield. Currently, crop breeding has entered biotechnology-based breeding involving transgenic, genome design, genome editing, and genomic breeding techniques. During the past decades, with the availability of reference genomes and advancement in sequencing technologies, genome-wide association studies (GWAS) and omics studies have been carried out in foxtail millet (Jia et al., 2013; Upadhyaya et al., 2015; Jaiswal et al., 2019), pearl millet (Varshney et al., 2017b), finger millet (Rahman et al., 2014; Sharma et al., 2018), cassava (Rabbi et al., 2017; Kayondo et al., 2018; Zhang et al., 2018; do Carmo et al., 2020), and orphan legume species such as pigeon pea (Varshney et al., 2017a), chickpea (Li et al., 2018), and common bean (Wu et al., 2020). Some of these crops used to be considered as orphan crop are now mainstream crops, and undergone genomic selection during past decades such as pearl millet (Varshney et al., 2017a), pea (Annicchiarico et al., 2017), cassava (Wolfe et al., 2017), pigeon pea (Bohra et al., 2020), peanut (Pandey et al., 2020), common bean (Keller et al., 2020), etc. Thus, there is need to employ application of genomic tools for genetic improvement of current orphan crops as well; so that they can be evolved as the mainstream crops with increased yield potential. Despite the knowledge gained through genomic studies, advancement in breeding has not accelerated in developing new improved varieties as expected. A more concerted genomics-assisted breeding approach is required for the genetic improvement of these orphan crops for yield and other agronomic traits. Details of cytogenetic and genomic information on selected orphan crops appear in Table 4.

**TABLE 4:** Cytogenetic and genome details of selected orphan crops.

Crop	Scientific name	Ploidy level	Chromosome number	Estimated genome size
Finger millet	*Eleusine coracana*	Allotetraploid	36	1.6 Gbp
Proso millet	*Panicum miliaceum*	Tetraploid	36	923 Mbp
Barnyard millet	*Echinochloa* sp.	Hexaploid	54	1.27 Gbp
Buckwheat	*Fagopyrum esculentum*	Diploid	16	540 Mbp
Fonio	*Digitaria exilis*	Tetraploid	36	893 Mbp
Little millet	*Panicum sumatrense*	Tetraploid	36	NA
African yam bean	*Sphenostylis stenocarpa*	Diploid	18, 20, 22, and 24	NA
African winged bean	(*Psophocarpus tetragonolobus*)	NA	NA	NA
Moth bean	*Vigna aconitifolia*	Diploid	22	NA
Bambara nut	*Vigna subterranea*	Diploid	20, 22	NA
Jatropha	*Jatropha curcas*	Diploid	22	339 Mbp
Jojoba	*Simmondsia chinensis*	Diploid	26	887 Mbp
Camelina	*Camelina sativa*	Hexaploid	40	785 Mb
Teff	*Eragrostis tef*	Allotetraploid	40	672 Mbp

## Policy Perspectives for Global Expansion of Orphan Crops

### Historical Perspective

Global agricultural systems are at a critical juncture. Agricultural policies, market incentives, trade forces, agricultural subsidies, and years of intensive research targeting agricultural productivity growth have skewed crop production portfolios toward a handful of high-yielding crops that can be grown to scale on the world’s mainstream agricultural lands (Tadele, 2019; Borelli et al., 2020). Policy support has been mainly directed at promoting the production and use of conventional crop species (e.g., wheat, rice, and maize) that dominate global food markets and underpin global diets that are becoming increasingly homogeneous (Borelli et al., 2020). Meanwhile, increasing evidence has come to light suggesting that the narrowing of diversity in crop species poses a significant threat to global food security and environmental sustainability (Khoury et al., 2014). From an environmental standpoint, these specialized modern production systems dominated by conventional crops require high-tech and high-input production systems, leading to the overexploitation of the natural resource base and ecosystems (Willett et al., 2019; Borelli et al., 2020). Given the high-level adaptation of orphan crop species to heterogeneous and harsh agro-ecological conditions, promoting orphan crops on a large scale could encourage the use of regenerative and ecological processes instead of relying entirely on intensification of conventional crops requiring a significant number of external inputs (i.e., chemicals and fertilizers) that are harmful to the environment. From the poverty elimination perspective, orphan crops are seen as “poor peoples’ crops,” which have the potential to not only enhance the food and nutrition security of the rural poor but also offer income-generating opportunities (Naluwairo, 2011). Investment in orphan crops could improve the livelihoods of socially marginal and vulnerable groups, including indigenous people and women (Egeland and Harrison, 2013; Azam-Ali et al., 2021).

In spite of the increased political will throughout recent years to promote investment in orphan crops, numerous studies generate evidence pointing to the lack of support for them, all of which recognize the need for change in the nature and scope of the policy environment (Mabhaudhi et al., 2017; Tadele, 2019; Tadele and Bartels, 2019; Kamenya et al., 2021). Previous literature on neglected crop species points to several impediments and challenges that limit the integration of orphan crops into commercial food systems (Fanzo et al., 2013). Inappropriate agriculture and food security policies that favor large cereal crops often diminish the dietary importance of orphan crops. There is still a lack of policies to support and recognize the nutritional value of neglected crops, which makes it difficult for locally produced traditional products to enter international marketplaces. There is very little research tying orphan crops to various diets and enhanced nutrition or comparing cost-effective strategies of using species for nutritional outcomes. Most of the essential data are dispersed and inaccessible through a single platform, which frequently goes unnoticed by policymakers. More crucial is the lack of infrastructure and markets nationally and regionally to channel neglected crop products to major food markets (Hunter et al., 2019; Kumar and Bhalothia, 2020).

### The Broader Policy Context: Global Policy Responses

*The State of the World’s Biodiversity for Food and Agriculture* highlighted a rapid decline and global disappearance of many local varieties of domesticated plants, many of which are neglected in local production systems and are mainly maintained by custodian farmers exclusively for subsistence and informal trade (FAO, 2019; Borelli et al., 2020). These plants nevertheless have a tremendous potential to redress key challenges in sustainable development, such as the vulnerability of production systems to climate change, disempowerment of vulnerable groups (women and indigenous peoples), widespread poverty, shrinking food biodiversity, and pervasive malnutrition (Egeland and Harrison, 2013; Azam-Ali et al., 2021). With the effects of climate change on the horizon posing critical challenges to agricultural systems, orphan crops have recently attracted increasing attention at the policy level because of their remarkable resilience to biotic and abiotic stresses and importance for rural food security, especially in marginal areas where production is constrained by biotic and socioeconomic constraints (Mabhaudhi et al., 2017).

In pursuit of achieving the Sustainable Development Goals (SDGs) related to the food security-poverty-environment nexus, the United Nations General Assembly (UNGA) has adopted several conventions to address the problems faced by global food production systems, including the lack of policy support for orphan and underused crops. The UN community has widely advocated investing in conserving these crop species and making greater use of the abundance and diversity of orphan crops in promoting sustainable diets (United Nations, 2019a,b). Subsequently, the UNGA conventions recognized several priority actions to transform production systems, especially to make underused and neglected crop species available to producers and consumers. These actions include directing increased research investments to explore their climate resilience and nutritional characteristics; providing greater support for their mainstreaming in food security policies and programs (e.g., public procurement); encouraging their use to diversify farming systems and create more biodiverse landscapes and healthier ecosystems; and upgrading their value chains and markets to ensure their sustainable use (FAO, 2019; United Nations, 2019a,b).

Similarly, the Second Global Plan of Action for Plant Genetic Resources for Food and Agriculture and the International Treaty on Plant Genetic Resources for Food and Agriculture lay out a series of agreed priority plans and actions that can protect the rich portfolio of diverse genetic resources, especially promoting the conservation and sustainable use of neglected and underused species (FAO, 2012). The Second Global Plan of Action aims to promote cost-efficient and effective global efforts to conserve and sustainably use orphan and neglected crops, link conservation with use for a greater use of plant germplasm, reinforce crop improvement and seed systems to foster economic development, create capacity, and strengthen national programs and widen partnerships for orphan crop management. The Second Global Plan of Action is a strategic framework that comprises 18 priority activities organized into four areas: (1) *in situ* conservation and management, (2) *ex situ* conservation, (3) sustainable use, and (4) sustainable institutional and human capacity. The agreed priority actions are instrumental in reorienting and prioritizing research and development (R&D) agendas.

Several national and international research and policy institutions have dedicated efforts to investigate and identify best management practices to improve orphan crops. Among others, these include CGIAR, the African Orphan Crops Consortium (AOCC), Crop Diversity Trust, Svalbard Global Seed Vault, Crops for the Future (CFF), and Tef Improvement Project. In addition, several funding agencies provide financial grants and aid for R&D of orphan crops, including the Bill & Melinda Gates Foundation (BMGF), McKnight Foundation, and Biotechnology and Biological Sciences Research Council (BBSRC) (Tadele, 2019). CGIAR plays a vital role among the international institutions involved in germplasm collection and conservation, but it focuses more on the major staple crops. Only a few of the 15 CGIAR centers are mandated to do R&D for orphan crops. The AOCC is an international effort to improve the nutrition, productivity, and climatic adaptability of some of Africa’s most important food crops; its work involves sequencing, assembling, and annotating the genomes of 100 traditional and underused African food crops, which will enable higher nutritional content for society over the decades to come (Jamnadass et al., 2020).

### The Way Forward: Research Engagement and Policy Priorities

**FIGURE 1:**
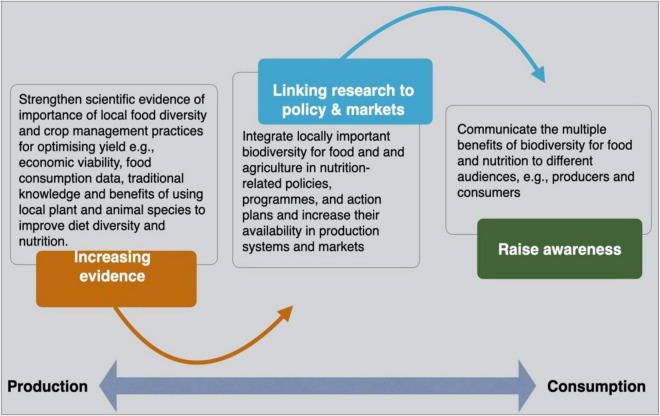
A three-pronged approach for mainstreaming underused and neglected crops. Source: Adopted from Borelli et al. (2020).

To put neglected crops back on the national agenda and for orphan crops to see a resurgence in local production systems and global food demand, strategic actions are required at different levels to mainstream orphan crops. These actions should target combining intensification with sustainable solutions (Tadele, 2017). A major part of the recent literature exploring producers’ and consumers’ behavior and preferences advocates for a three-pronged approach to establish enabling environments for mainstreaming neglected crops for food and nutrition in local food systems (Hunter et al., 2019; Borelli et al., 2020; McMullin et al., 2021). This three-pronged approach (Figure 1) revolves around three avenues of R&D activities: (1) generating increased evidence about the nutritional value and biocultural importance of neglected crops, (2) linking research to policy and strategic plans to ensure that underused crop species are considered in national food and nutrition security strategies and actions, and (3) improving the knowledge base to increase consumer awareness about the nutritional desirability of underused crops to incorporate these crops into their food systems, markets, and diets.

The three-pronged strategy to investigate orphan crop mainstreaming solutions reveals innovative research pathways for examining producer and consumer behavior and preferences. Further research is essential to broaden our understanding of the incentives and factors that influence diverse groups of farmers’ productive activities (Gassner et al., 2019; Borelli et al., 2020; McMullin et al., 2021). It is necessary to take into account a considerably broader range of additional aspects, including long-term aspirations of the farming communities (Mausch et al., 2018). In order for orphan crop foods to compete more effectively with other food crops in more complex market-based food systems, a deeper knowledge of consumers’ preferences and behavior in relation to orphan food crops is required (Revoredo-Giha et al., 2018). These must be considered in relation to the consumers’ age, education, income, location, and media access. Because of the importance of orphan crops in traditional food systems, it is also necessary to analyze and understand consumers’ cultural backgrounds (McMullin et al., 2021).

Future strategies should highlight and promote the design and execution of a variety of interventions aimed at enhancing and improving value chains for neglected crops. The development of quality assurance measures to indicate enhanced nutritional value, demand creation through marketing campaigns and food product development, supply chain development to provide planting materials to farmers, and stimulating private sector involvement to create “shared values” are some examples (Waized et al., 2015). Orphan or indigenous crops are associated with some desirable properties that can be useful in the elevation of the crops to a higher rate of production (Tadele, 2019). However, low yield remains a challenge for producers and policymakers to take orphan crops to the next level. The application of modern genetic and genomic tools to the breeding of these crops can provide enormous opportunities for ensuring world food security (Tadele, 2018). Technological developments in terms of breeding and varietal development should be tailored to the context of harsh and fragile environments where orphan crops can strive. It is also vital to strengthen the seed system for orphan crops to maintain local production systems. Public-private partnerships in the multiplication and distribution of better seed to marginal and isolated agricultural production locations should be encouraged through breeding programs.

With the impending effects of climate change, abiotic stresses such as drought, salinity, and heat, as well as climate change, have a significant impact on crop yield and food security. Future research should focus on creating resistance to or tolerance of these environmental stresses. Several orphan crops are nutrient-dense and their ability to adapt to harsh conditions suggests that they can be deployed as part of efforts to champion climate-change adaptation, improved agriculture, and economic advancement for smallholder farmers residing in low-production environments. However, some of the crops’ negative characteristics (for example, low production) must be improved (Tadele, 2019). Future policies must integrate intensification with sustainable solutions (Tadele, 2017). Future agricultural policies targeting orphan crops should also encourage agro-ecological practices by encouraging the use of orphan crops in operations with the goal of decreasing external inputs and maximizing plant hardiness in vulnerable, drought-prone areas where underused crops can exploit residual soil moisture and scarce rainfall.

Building social capital can help farming communities and grower associations strengthen their governance and technical capacity, particularly for indigenous farmers with limited access to information. Strengthening the capacity of farming communities including women, youth, and indigenous people is fundamental for boosting production efficiency, along with improved post-production, technologies, business and entrepreneurship skills, markets and market information, and sustainable investments in physical infrastructure. This could be pursued as part of a larger public-private partnership initiative and investment goal in agricultural marketing of orphan crops.

Local and indigenous knowledge should be valued as a vital instrument in future policies for agriculture and food security and should be carefully considered and documented, when possible, with the cooperation of indigenous and local populations. Indigenous farmers who typically grow traditional and neglected crops are losing valuable expertise about these crops; thus, traditional knowledge linked with orphan crops is in grave danger of eroding and disappearing. Traditional knowledge on orphan crops is critical not only for the rural farmers who produce them but also for scientific study on crop improvement in general. Future policies should encourage investing in the preservation and protection of traditional knowledge.

## Conclusion

Orphan crops have undeniable health and nutritional benefits, ability to cope with harsh and suboptimal growing conditions, and broad ecosystem suitability. Thus, they are proven crops with enormous potential to combat food and nutrition insecurity, enrich and diversify diets and crop production systems, improve farmers’ livelihood, as well as use and improve degraded soils in marginal environments. Complementary mainstreaming of these crops into production systems, genetic enhancement, and continuous improvement of the crops for stress tolerance and efficient use of resources through modern breeding approaches, coupled with the use of appropriate agronomic practices, will contribute immensely to increased global crop production. Increased awareness of the importance of orphan crops among stakeholders and favorable policies to provide the crops with required attention, opportunities, and competitive advantage will go a long way to addressing the global food deficit and the challenges of malnutrition.

## Author Contributions

RS conceived the idea for the manuscript. AT, PV, ST, HR, HA, NN, MS, and RS drafted and revised the manuscript. All authors agreed to the final submitted version of the manuscript.

## Conflict of Interest

The authors declare that the research was conducted in the absence of any commercial or financial relationships that could be construed as a potential conflict of interest.

## Publisher’s Note

All claims expressed in this article are solely those of the authors and do not necessarily represent those of their affiliated organizations, or those of the publisher, the editors and the reviewers. Any product that may be evaluated in this article, or claim that may be made by its manufacturer, is not guaranteed or endorsed by the publisher.

## Footnotes

1
http://africanorphancrops.org/

2
https://www.iita.org/news-item/raising-profile-yam-whole-genome-sequencing-neglected-orphan-crop/
