# Supplementary material for: Orphan Crops: A Best Fit for Dietary Enrichment and Diversification in Highly Deteriorated Marginal Environments
Source: Front Plant Sci. 2022 Feb 24;13:839704. doi: 10.3389/fpls.2022.839704 (PMC8908242; doi:10.3389/fpls.2022.839704)
Supplement: Supplementary file 1 [file Table_1.DOC]

**Supplementary Table 1: Crop-wise details on collections of selected orphan crops**

| **Holding institute** | **Country** | **WIEWS Code** | | **Total number of accessions** | | **Origin of germplasm** | | | **Landraces /Traditional cultivar** | **Wild relatives** | **Advanced/improved varieties/cultivar** | **Breeding/Research materials** | **Others** | **Source of information** |
| --- | --- | --- | --- | --- | --- | --- | --- | --- | --- | --- | --- | --- | --- | --- |
|  |  |  |  |  |  | Indigenous | Exotic | |  |  |  |  |  |  |
| **Finger millet (*Eleusine coracana*)** | | | | | | | | | | | | | | |
| International Crop Research Institute for the Semi-Arid Tropics (ICRISAT) | India | IND002 | | 7,519 | | 1,434 | 5650 | | 7121 | 205 | 50 | 143 |  | GENESYS |
| Genetic Resources Research Institute (GeRRI) | Kenya | KEN212 | | 2,934 | | 1,910 | 1,024 | | 1,858 | 30 |  | 20 | 21 | GENESYS |
| Plant Genetic Resources Conservation Unit, Southern Regional Plant Introduction Station, University of Georgia, USDA-ARS | USA | USA016 | | 738 | |  | 738 | |  |  | 1 |  | 737 | USDA National Plant Germplasm System |
| The National Lab for Genetic Resources Preservation (NLGRP)National Center for Genetic Resources Preservation | USA | USA995 | | 702 | | 5 | 697 | |  |  | 1 |  | 701 | USDA National Plant Germplasm System |
| SADC Plant Genetic Resources Centre | Zambia | ZMB030 | | 701 | | 353 | 348 | | 697 |  |  |  |  | GENESYS |
| International Center for Biosaline Agriculture (ICBA) | UAE | ARE003 | | 80 | |  | 80 | | 80 |  |  |  |  | INHOUSE |
| AGES Linz - Austrian Agency for Health and Food Safety / Seed Collection | Austria | AUT001 | | 10 | |  | 10 | | 10 |  |  |  |  | EURISCO |
| Gene bank — Czechia | Czechia | CZE122 | | 1 | |  |  | |  |  | 1 |  |  | GENESYS |
| International Livestock Research Institute (ILRI) | Ethiopia | ETH013 | | 1 | | 1 |  | |  | 1 |  |  |  | GENESYS |
| Millennium Seed Bank Project, Seed Conservation Department, Royal Botanic Gardens, Kew, Wakehurst Place | United Kingdom | GBR004 | | 1 | |  | 1 | |  |  |  |  | 1 | GENESYS |
| **Proso millet (*Panicum miliaceum*)** | | | | | | | | | | | | | | |
| International Crop Research Institute for the Semi-Arid Tropics (ICRISAT) | India | IND002 | 849 | | 22 | | |  | 812 | 1 | 8 | 4 | 2 | GENESYS |
| Istituto di Bioscienze e Biorisorse, Consiglio Nazionale delle Ricerche ((IBBR-CNR)) | Italy | ITA436 | 16 | |  | | | 16 | 4 |  |  |  |  | EURISCO |
| Genebank Tyrol / Tyrolean Government | Austria | AUT005 | 8 | | 1 | | | 1 | 1 |  | 3 | 4 |  | GENESYS |
| Gene bank | Czechia | CZE122 | 4 | | 1 | | |  |  |  | 1 |  |  | GENESYS |
| Genetic Resources Research Institute (GeRRI) | Kenya | KEN212 | 4 | | 4 | | |  | 4 |  |  |  |  | GENESYS |
| Genetic Resources Unit, Institute of Biological, Environmental & Rural Sciences, Aberystwyth University | United Kingdom | GBR016 | 3 | |  | | |  |  |  | 1 |  | 2 | GENESYS |
| AGES Linz - Austrian Agency for Health and Food Safety / Seed Collection | Austria | AUT001 | 2 | | 1 | | | 1 | 1 | 1 |  |  |  | GENESYS |
| Institute for Seed and Seedlings | Croatia | HRV053 | 2 | | 1 | | | 1 |  |  | 2 |  |  | GENESYS |
| Office of the Styrian Regional Government, Department for Plant Health and Special Crops | Austria | AUT025 | 1 | | 1 | | |  |  |  | 1 |  |  | GENESYS |
| Portuguese Bank of Plant Germplasm | Portugal | PRT001 | 1 | | 1 | | |  | 1 |  |  |  |  | GENESYS |
| **Barnyard millet (*Echinochloa spp*.)** | | | | | | | | | | | | | | |
| International Crop Research Institute for the Semi-Arid Tropics (ICRISAT) | India | IND002 | 749 | | 453 | | | 198 | 749 |  |  |  |  | GENESYS |
| North Central Regional Plant Introduction Station, USDA-ARS, NCRPIS | USA | USA020 | 316 | | 31 | | | 278 |  | 40 | 4 | 2 | 270 | USDA National Plant Germplasm System |
| Genetic Resources Research Institute (GeRRI) | Kenya | KEN212 | 210 | | 207 | | | 3 |  | 18 |  |  |  | GENESYS |
| International Center for Biosaline Agriculture (ICBA) | UAE | ARE003 | 145 | |  | | | 124 |  | 1 |  |  | 144 | IN HOUSE |
| International Livestock Research Institute (ILRI) | Ethiopia | ETH013 | 91 | | 13 | | | 69 |  | 90 | 1 |  |  | GENESYS |
| Australian Grains Genebank, Department of Economic Development Jobs Transport and Resources | Australia | AUS165 | 69 | | 2 | | | 58 |  |  |  |  |  | GENESYS |
| Millennium Seed Bank Project, Seed Conservation Department, Royal Botanic Gardens, Kew, Wakehurst Place | United Kingdom | GBR004 | 55 | | 1 | | | 52 |  |  |  |  | 55 | GENESYS |
| Genebank, Leibniz Institute of Plant Genetics and Crop Plant Research | EURISCO | DEU146 | 36 | | 6 | | | 30 | 5 | 5 |  | 3 | 2 | GENESYS |
| Australian Pastures Genebank (APG) | Australia | AUS167 | 25 | | 2 | | | 10 |  | 25 |  |  |  | GENESYS |
| Centro Internacional de Agricultura Tropical (CIAT) | Colombia | COL003 | 20 | |  | | | 20 |  | 20 |  |  |  | GENESYS |
| **Buckwheat millet (*Fagopyrum tataricum)*** | | | | | | | | | | | | | | |
| N.I. Vavilov Research Institute of Plant Industry (VIR) | Russia | RUS001 | 2,110 | | 895 | | | 1,213 |  |  |  |  |  | EURISCO |
| Ustymivka Experimental Station of Plant Production | Ukraine | UKR008 | 1,457 | | 843 | | | 609 | 1,084 |  | 102 | 144 |  | EURISCO |
| Podil's'ka State Agrarian-Technical Academy | Ukraine | UKR130 | 555 | | 334 | | | 197 | 38 |  | 43 | 2 |  | EURISCO |
| Plant Breeding and Acclimatization Institute (IHAR) | Poland | POL003 | 202 | | 10 | | | 13 | 13 |  | 9 |  |  | EURISCO |
| Agronomy Department, Biotechnical Faculty, University of Ljubljana | Slovenia | SVN018 | 186 | | 95 | | |  | 10 |  | 142 |  |  | EURISCO |
| Northeast Regional Plant Introduction Station, Plant Genetic Resources Unit, USDA-ARS, New York State Agricultural Experiment Station, Cornell University | USA | USA003 | 178 | | 36 | | | 106 |  | 1 | 3 |  | 170 | USDA National Plant Germplasm System |
| Institute for Agrobotany | Hungary | HUN003 | 174 | | 31 | | | 29 | 43 |  | 37 |  |  | EURISCO |
| Gene bank — Czechia | Czechia | CZE122 | 128 | | 1 | | | 102 | 5 |  | 55 |  |  | EURISCO |
| Genebank, Leibniz Institute of Plant Genetics and Crop Plant Research | Germany | DEU146 | 100 | | 3 | | | 81 | 5 |  | 55 |  |  | EURISCO |
| Embrapa Recursos Genéticos e Biotecnologia | Brazil | BRA003 | 77 | |  | | | 61 | 66 | 3 | 25 |  |  | GENESYS |
| **Fonio (*Digitaria* sp.)** | |  | | | | | | | | | | | | |
| Australian Pastures Genebank | Australia | AUS167 | 17 | | 8 | | | 9 |  | 16 | 1 |  |  | GENESYS |
| International Livestock Research Institute | Ethiopia | ETH013 | 23 | |  | | | 23 |  | 23 |  |  |  | GENESYS |
| Millennium Seed Bank Project, Seed Conservation Department, Royal Botanic Gardens, Kew, Wakehurst Place | United Kingdom | GBR004 | 1 | |  | | | 1 |  |  |  |  | 1 | GENESYS |
| National Centre for Genetic Resources and Biotechnology | Nigeria | NGA010 | 1 | |  | | |  | 1 |  |  |  |  | GENESYS |
| Plant Genetic Resources Conservation Unit, Southern Regional Plant Introduction Station, University of Georgia, USDA-ARS | USA | USA016 | 2 | |  | | | 2 |  |  |  |  | 2 | USDA National Plant Germplasm System |
| **Little millet (*Panicum sumatrense*)** | | | | | | | | | | | | | | |
| International Crop Research Institute for the Semi-Arid Tropics (ICRISAT) | India | IND002 | 473 | | 466 | | | 7 | 473 |  |  |  |  | GENESYS |
| North Central Regional Plant Introduction Station, USDA-ARS, NCRPIS | USA | USA020 | 211 | |  | | | 211 |  |  |  |  | 211 | USDA National Plant Germplasm System |
| International Livestock Research Institute | Ethiopia | ETH013 | 7 | |  | | | 2 |  | 4 | 3 |  |  | GENESYS |
| Australian Pastures Genebank | Australia | AUS167 | 3 | | 3 | | |  |  | 2 | 1 |  |  | GENESYS |
| AGES Linz - Austrian Agency for Health and Food Safety / Seed Collection | Austria | AUT001 | 1 | |  | | | 1 |  | 1 |  |  |  | EURISCO |
| Millennium Seed Bank Project, Seed Conservation Department, Royal Botanic Gardens, Kew, Wakehurst Place | United Kingdom | GBR004 | 1 | |  | | | 1 |  |  |  |  | 1 | GENESYS |
| Institute of Plant Production n.a. V.Y. Yurjev of UAAS | Ukraine | UKR001 | 1 | |  | | | 1 |  |  | 1 |  |  | GENESYS |
| **African yam bean (*Sphenostylis stenocarpa*)** |  |  |  | |  | | |  |  |  |  |  |  |  |
| International Institute of Tropical Agriculture (IITA) | Nigeria | NGA039 | 495 | | 335 | | | 9 | 349 |  | 2 |  | 144 | GENESYS |
| Plant Genetic Resources Conservation Unit, Southern Regional Plant Introduction Station, University of Georgia, USDA-ARS | USA | USA016 | 12 | |  | | | 12 |  |  |  |  | 12 | USDA National Plant Germplasm System |
| Botanic Garden Meise | Belgium | BEL014 | 1 | |  | | |  |  | 1 |  |  |  | EURISCO |
| Millennium Seed Bank Project, Seed Conservation Department, Royal Botanic Gardens, Kew, Wakehurst Place | United Kingdom | GBR004 | 1 | |  | | | 1 |  |  |  |  | 1 | GENESYS |
| **African winged bean (*Psophocarpus tetragonolobus*)** |  |  |  | |  | | |  |  |  |  |  |  |  |
| World Vegetable Center | Taiwan | TWN001 |  | | 5 | | | 281 | 132 | 11 | 1 | 5 | 138 | GENESYS |
| Plant Genetic Resources Conservation Unit, Southern Regional Plant Introduction Station, University of Georgia, USDA-ARS | USA | USA016 | 174 | | 77 | | |  |  |  | 1 |  | 173 | USDA National Plant Germplasm System |
| Australian Grains Genebank, Agriculture Victoria | Australia | AUS165 | 170 | |  | | | 163 |  |  |  |  | 170 | GENESYS |
| International Institute of Tropical Agriculture | Nigeria | NGA039 | 55 | | 8 | | | 22 | 19 |  |  |  | 36 | GENESYS |
| Centro Agronómico Tropical de Investigación y Enseñanza | Costa Rica | CRI001 | 16 | | 6 | | | 10 | 16 |  |  |  |  | GENESYS |
| Embrapa Recursos Genéticos e Biotecnologia | Barzil | BRA003 | 15 | | 4 | | |  |  |  |  |  | 15 | GENESYS |
| International Livestock Research Institute | Ethiopia | ETH013 | 12 | |  | | | 4 |  | 11 | 1 |  |  | GENESYS |
| Genetic Resources Research Institute | Kenya | KEN212 | 10 | |  | | | 10 |  |  |  |  | 10 | GENESYS |
| Desert Legume Program | USA | USA971 | 4 | | 1 | | |  |  |  |  |  | 4 | USDA National Plant Germplasm System |
| Institute for Plant Genetic Resources 'K.Malkov' | Bulgaria | BGR001 | 2 | |  | | |  |  |  |  |  | 2 | EURISCO |
| **Amaranthus (*Amaranth spp*.)** | | | | | | | | | | | | | | |
| North Central Regional Plant Introduction Station, USDA-ARS, NCRPIS | USA | USA020 | 3,342 | | 278 | | | 2993 |  | 349 | 1638 |  | 1194 | USDA National Plant Germplasm System |
| Embrapa Recursos Genéticos e Biotecnologia | Brazil | BRA003 | 2,495 | | 11 | | | 848 |  |  |  |  |  |  |
| World Vegetable Center (WorldVeg) | Taiwan | TWN001 | 790 | | 13 | | | 636 | 127 | 75 |  |  | 3 | GENESYS |
| Institute for Agrobotany | Hungary | HUN003 | 787 | | 37 | | | 84 | 30 | 22 | 2 | 19 |  | EURISCO |
| International Center for Biosaline Agriculture (ICBA) | UAE | ARE003 | 415 | | 2 | | | 296 | 413 |  |  |  | 2 | IN HOUSE |
| Centro Agronómico Tropical de Investigación y Enseñanza | Costa Rica | CRI001 | 265 | | 2 | | | 263 | 227 | 15 |  |  |  | GENESYS |
| Millennium Seed Bank Project, Seed Conservation Department, Royal Botanic Gardens, Kew, Wakehurst Place | United Kingdom | GBR004 | 243 | | 8 | | | 224 |  |  |  |  | 32 | GENESYS |
| Australian Grains Genebank, Department of Economic Development Jobs Transport and Resources | Australia | AUS165 | 215 | | 1 | | | 203 |  |  |  |  |  | GENESYS |
| Genetic Resources Research Institute (GeRRI) | Kenya | KEN212 | 209 | | 195 | | | 14 | 74 | 44 | 14 |  |  | GENESYS |
| Genebank, Leibniz Institute of Plant Genetics and Crop Plant Research | Germany | DEU146 | 159 | | 2 | | | 72 | 89 | 36 | 14 |  |  | GENESYS |
| **Moth bean (*Vigna aconitifolia*)** | | | | | | | | | | | | | | |
| N.I. Vavilov Research Institute of Plant Industry (VIR) | Russia | RUS001 | 64 | | 1 | | | 50 | 64 |  |  |  |  | EURISCO |
| Plant Genetic Resources Conservation Unit, Southern Regional Plant Introduction Station, University of Georgia, USDA-ARS | USA | USA016 | 58 | |  | | | 58 |  |  |  |  | 58 | USDA National Plant Germplasm System |
| Genetic Resources Research Institute (GeRRI) | Kenya | KEN212 | 50 | | 1 | | | 49 | 24 |  |  |  |  | GENSYS |
| Australian Grains Genebank, Department of Economic Development Jobs Transport and Resources | Australia | AUS167 | 35 | |  | | | 34 |  |  |  |  |  | GENSYS |
| World Vegetable Center (WorldVeg) | Taiwan | TWN001 | 28 | |  | | | 27 |  | 1 |  |  |  | GENSYS |
| Ustymivka Experimental Station of Plant Production | Ukraine | UKR008 | 27 | |  | | | 27 | 27 |  |  |  |  | EURISCO |
| Botanic Garden Meise | Belgium | BEL014 | 19 | |  | | | 18 | 19 |  |  |  |  | EURISCO |
| Embrapa Recursos Genéticos e Biotecnologia | Brazil | BRA003 | 7 | | 6 | | |  |  |  | 2 |  |  | GENESYS |
| Genebank, Leibniz Institute of Plant Genetics and Crop Plant Research | Brazil | DEU146 | 7 | |  | | | 6 | 4 |  |  |  |  | GENESYS |
| **Bambara nut (*Vigna subterranea*)** | | | | | | | | | | | | | | |
| International Institute of Tropical Agriculture | Nigeria | NGA039 | 1904 | | 321 | | | 1054 | 73 | 43 | 1030 |  | 758 | GENESYS |
| SADC Plant Genetic Resources Centre | Zambia | ZMB030 | 166 | | 83 | | | 85 | 166 |  |  |  |  | GENESYS |
| Agricultural Plant Genetic Resources Conservation and Research Centre | Sudan | SDN002 | 36 | | 36 | | |  | 34 | 1 |  |  |  | GENESYS |
| International Livestock Research Institute | Ethiopia | ETH013 | 17 | |  | | | 17 |  | 17 | ­­ |  |  |  |
| Genetic Resources Research Institute | Kenya | KEN212 | 12 | | 10 | | | 2 | 12 |  |  |  |  |  |
| **Jojoba (*Simmondsia chinensis*)** | | | | | | | | | | | | | | |
| National Arid Land Plant Genetic Resources Unit, USDA, ARS | USA | USA955 | 270 | | 54 | | |  | 111 | 2 |  |  | 212 | USDA National Plant Germplasm System |
| International Center for Biosaline Agriculture | UAE | ARE003 | 29 | | 29 | | |  | 29 |  |  |  |  | INHOUSE |
| Australian Pastures Genebank | Australia | AUS167 | 8 | |  | | |  |  | 2 |  |  | 2 | GENESYS |
| Australian Grains Genebank, Agriculture Victoria | Australia | AUS165 | 1 | |  | | | 1 |  |  |  |  | 1 | GENESYS |
| Institute for Plant Genetic Resources 'K.Malkov' | Bulgaria | BGR001 | 1 | |  | | |  |  |  |  |  | 1 | EURISCO |
| Millennium Seed Bank Project, Seed Conservation Department, Royal Botanic Gardens, Kew, Wakehurst Place | United Kingdom | GBR004 | 1 | |  | | | 1 |  |  |  |  | 1 | GENESYS |
| Genetic Resources Research Institute | Kenya | KEN212 | 1 | |  | | | 1 |  |  |  |  | 1 | GENESYS |
| **Jatropha (*Jatropha curcas*)** | | | | | | | | | | | | | | |
| Genetic Resources Unit, ICRAF | Kenya | KEN056 | 460 | | 20 | | | 440 |  | 454 |  |  | 6 | GENESYS |
| Embrapa Algodão | Brazil | BRA007 | 104 | |  | | |  |  |  |  |  |  | GENESYS |
| Embrapa Recursos Genéticos e Biotecnologia | Brazil | BRA003 | 35 | |  | | |  |  |  |  |  |  | GENESYS |
| Millennium Seed Bank Project, Seed Conservation Department, Royal Botanic Gardens, Kew, Wakehurst Place | UK | GBR004 | 5 | |  | | | 4 |  |  |  |  | 5 | GENESYS |
| Genetic Resources Research Institute (GeRRI) | Kenya | KEN212 | 4 | | 4 | | |  |  | 4 |  |  |  | GENESYS |
| Australian Pastures Genebank (APG) | Australia | AUS167 | 2 | |  | | | 2 |  | 2 |  |  |  | GENESYS |
| Centro Agronómico Tropical de Investigación y Enseñanza | Costa Rica | CRI001 | 2 | |  | | | 2 | 2 |  |  |  |  | GENESYS |
| Tropical Agricultural Research Station, Clonal Repository USDA/ARS | USA | USA108 | 1 | | 1 | | |  |  |  |  |  | 1 | USDA National Plant Germplasm System |
| **Camelina (*Camelina sativa*)** | | | | | | | | | | | | | | |
| Genebank, Leibniz Institute of Plant Genetics and Crop Plant Research | Germany | DEU146 | 249 | | 109 | | | 66 | 6 | 44 | 47 | 70 | 84 | GENESYS |
| Department of Crop Sciences, University of Natural Resources and Life Sciences | Austria | AUT007 | 200 | | 67 | | | 130 | 13 |  | 31 |  | 46 | EURISCO |
| Plant Breeding and Acclimatization Institute | Poland | POL003 | 96 | | 8 | | | 10 | 2 |  | 4 |  |  | EURISCO |
| Gene bank — Czechia | Czechia | CZE122 | 89 | |  | | | 86 | 3 | 16 | 34 | 35 |  | EURISCO |
| Australian Grains Genebank, Agriculture Victoria | Australia | AUS165 | 59 | | 1 | | | 44 |  |  |  |  | 59 | GENESYS |
| Institute for Plant Genetic Resources 'K.Malkov' | Bulgaria | BGR001 | 71 | | 1 | | | 35 | 1 |  | 5 |  |  | EURISCO |
| Nordic Genetic Resource Center | Sweden | SWE054 | 49 | | 1 | | | 48 |  |  | 1 | 48 |  | EURISCO |
| North Central Regional Plant Introduction Station, USDA-ARS, NCRPIS | USA | USA020 | 30 | | 3 | | |  | 5 | 4 | 5 |  |  | USDA National Plant Germplasm System |
